# Cadmium Phytoremediation by *Arundo donax* L. from Contaminated Soil and Water

**DOI:** 10.1155/2013/324830

**Published:** 2013-12-31

**Authors:** Maria Sabeen, Qaisar Mahmood, Muhammad Irshad, Iftikhar Fareed, Afsar Khan, Farid Ullah, Jamshaid Hussain, Yousaf Hayat, Sobia Tabassum

**Affiliations:** ^1^Department of Environmental Sciences, COMSATS Institute of Information Technology, Abbottabad 22060, Pakistan; ^2^Department of Natural Resource Engineering and Management, University of Kurdistan Hewlêr, Kurdistan, Iraq; ^3^Department of Chemistry, COMSATS Institute of Information Technology, Abbottabad, Pakistan; ^4^Department of Mathematics, Statistics and Computer Sciences, KPK Agricultural University, Peshawar, Pakistan; ^5^Interdisciplinary Research Centre in Biomedical Materials, COMSATS Institute of Information Technology, Defence Road, Off Raiwind Road, Lahore, Pakistan

## Abstract

The potential of *Arundo donax* L. for phytoextraction of cadmium (Cd) from contaminated soil and water was probed. The plants were grown under greenhouse conditions in pots containing a nutrient solution or soil with increasing doses of Cd (0, 50, 100, 250, 500, 750, and 1000 **μ**g L^−1^) for 21 days. The growth and physiology of plants were evaluated at the end of the experiment. The maximum Cd content in root was 300 **μ**g g^−1^ during hydroponics experiments over 230 **μ**g g^−1^ in soil experiment. Cd concentration in stem was 262 **μ**g g^−1^ at 750 **μ**g L^−1^ supplied Cd in hydroponics over 191.2 **μ**g g^−1^ at 1000 in soil experiment. The maximum Cd concentration in leaves from hydroponics was 187 **μ**g g^−1^. Relatively low Cd uptake occurred during soil experiment with low translocation factor (TF) values. Both Bioaccumulation Factor (BF) and TF values for hydroponics were greater than 1. The IC_50_ values of ABTS and DPPH showed that both time and increasing Cd concentrations affected the production of antioxidants with lower half maximal inhibitory concentration (IC_50_) value on the 21st days. *A. donax* showed better potential for Cd remediation of aquatic environments.

## 1. Introduction

Consequent to global industrialization, heavy metal pollution is a widespread problem which has become a major environmental concern due to hazardous effects on human and environmental health. In industrialized societies, heavy metals are the world over environmental contaminants. Air or water pollution by metals varies from soil pollution, because heavy metals persevere in soil for a longer time period as compared with the other compartment of the biosphere [1]. In the latest decades, the yearly global release of heavy metals attained 22,000 t (metric ton) for cadmium, 939,000 t for copper, 783,000 t for lead, and 1,350,000 t for zinc [2]. Cadmium (Cd), a highly toxic metal pollutant of soils, inhibits root and shoot growth and yield production, affects nutrient uptake and homeostasis, is frequently accumulated by agriculturally important crops, and then enters the food chain with a significant potential to impair animal and human health [3]. The mutagenic aptitude of toxic heavy metals causes DNA damage and probably causes carcinogenic effects in animals and humans [4]. The prime health perils coupled with Cd are damage to the neurological system, having indications like uncontrollable shaking, muscle wasting, partial blindness, and deformities in children exposed in the womb [5].

Many phytoremediation technologies have been used for the remediation of polluted soils and water throughout the World [6]. Phytoremediation costs almost one-fourth of the other physical and chemical methods of pollutant treatment. The major advantages of the process include: improvement of the soil quality, as it is driven by solar energy thus suitable to most regions and climates, cost effective and technically feasible process, plants serve as sufficient biomass for rapid remediation; promote high rhizosphere activity and finally restoration in a reasonable time frame of 2 to 3 years.

The use of low cost, fast growing indigenous plants with efficient biomass producing plant species such as *Arundo donax* L. is highly desirable for phytoremediation of metal contaminated sites and waters. It is cultivable throughout Asia, Southern Europe, North Africa, and the Middle East for thousands of years with local names of “Giant Reed,” “Nurr,” “Nurru,” or “Nurro” [7–9]. It is considered as one of the most biologically productive of all communities [10]. Previous experiments on giant reed suggested that the stem height and diameter, number of nodes, fresh and dry weight of leaves, and net photosynthesis were not affected, indicating that plants tolerated the high concentrations of Cd and Ni [11]. As giant reed plants are very promising energy plants, they can be cultivated in contaminated soils to provide biomass for energy production purposes [11]. Our research group is exploring the potential of this plant in environmental remediation of various heavy metals at high concentrations. The plant showed some potential against arsenic [7–9] and chromium [10] remediation. The specific objective of the current study was to investigate the phytoremediation ability of *A. donax* for cadmium remediation and to compare the Cd removal from contaminated soil and water.

## 2. Materials and Methods

### 2.1. Plant Material

The plant material used for the present study was *A. donax* L. with the aim to evaluate its responses to cadmium added to liquid medium. The plant material was collected near PMA road Abbottabad, Khyber Pakhtunkhwa, Pakistan. Plants were collected for the heavy metal analysis. The soil-grown plants used for hydroponics tests were taken from growth of young meristematic buds grown in sterile aqueous medium [8]. The young plants were transplanted in plastic trays containing 1/2 strength basal nutrient solutions.

### 2.2. Experimental Design

The phytoremediation ability of *A. donax* was compared in Cd contaminated soil and aqueous solution. The Cd containing aqueous solution was prepared by dissolving cadmium chloride salt in the double distilled water. Various Cd treatments were given in triplicates to experimental plants (both in hydroponics and soil). Cd treatments included 0 (control), 50, 100, 250, 500, 750, and 1000 *μ*g L^−1^. Each pot contained 4 *A. donax* L. plants with an average 250 g biomass on fresh weight basis. Two kinds of experiments were performed in randomized complete block design (RCBD) with three replications for plants grown in Hoagland solution [12] and in soil. Plants with healthy and uniform shoots of almost equal morphological characteristics were immersed in nutrient solution for three weeks. Plants were grown under greenhouse conditions in the laboratory with Hoagland solution continuously aerated and renewed after every 2-3 days with an addition of 100–200 mL of nutrient solution.  Table 1 shows the average values of various plant characteristics.

### 2.3. Growth Parameters

Various growth parameters of *A. donax* were studied under cadmium stress including plant height (cm), number of leaves per plant, number of nodes per plant, dry weight, average root length (cm), and toxicity symptoms prior to and after the cadmium treatments.

### 2.4. Physiological Parameters

The physiological parameters of the plants included photosynthetic pigments, and the determination of antioxidants.

#### 2.4.1. Photosynthetic Pigments

At the time of harvest, fresh leaves of both of various treatments from both kinds of experiments were collected for the determination of chlorophyll a, chlorophyll b, and carotene contents. Leaves were cut into small pieces and 0.5 g of the sample was put into the glass test tubes. Then 10 mL of 80% acetone was added to the tubes and kept overnight for complete extraction. Photosynthetic pigments were determined spectrophotometrically using the visible wavelengths of 663, 645, and 480 nm for chlorophyll a, chlorophyll b and carotene, respectively [12].

#### 2.4.2. Antioxidant Determination

To determine antioxidant activity another parallel set of experiment was conducted. In this experiment four replicates each of 250 gm against each treatment was planted in a pot. Each pot was given Hoagland's solution and the respective treatment of cadmium above mentioned. After each week, one set was taken out and new leaves were counted. These plants were placed outside for shade dry.

All the plants were ground until uniform size. Now this sample was dipped in methanol for 4 to 5 days. The plant extract was filtered and methanol was recovered by passing it through rotary. The thick left over extract was taken out in Petri dishes and placed in fume hood. The thick jelly like extract was stored in sample bottles for further analysis.

*DPPH Free Radical Scavenging Assay*. 2,2-diphenyl-1-picrylhydrazyl free radical (DPPH) scavenging activity of crude extract and various fractions were estimated by standard DPPH assay protocol with certain modifications [13]. The reaction mixture contains 0.5 mL of test samples and 2.5 mL of DPPH in methanol. Concentration of DPPH was 100 *μ*M in the reaction mixture. These reaction mixtures were incubated for 30 min. at 37°C. The absorbance was measured at 517 nm using spectrophotometer (5000, Irmeco GmbH D-21496 Geesthacht, Germany). Percent radical scavenging activity or percent antioxidant index (AI%) by sample treatment was determined by comparison with methanol treated control group. An IC_50_ value denotes the concentration of sample, which is required to scavenge 50% DPPH free radicals. Propyl gallate was used as positive controls. 

*ABTS* ^*+*^ *Assay*. Total antioxidant activity was evaluated applying an improved 2,2′-azinobis-3-ethyl-benzothiazoline-6-sulfonic acid cation (ABTS) decolorization assay by Re et al. [14]. ABTS^+^ radical cation (ABTS^**+**^) was produced by reacting ABTS stock solution (7 mM) with 2.45 mM potassium persulphate and allowing the solution to stand in the dark at room temperature for 12–14 hours before use. For the study of total antioxidant activity, the solution was diluted with ethanol to an absorbance of 0.70 (±0.02) at 374 nm. Percentage inhibition was calculated by using the following equation:

(1) $$\begin{array}{c} \text{Percentage}\;\text{inhibition}=\left[1-\frac{{\text{absorbance}}_{\text{sample}}}{{\text{absorbance}}_{\text{control}}}\right]\times 100. \end{array}$$

IC_50_ values were calculated based on various determinations of these antioxidants by supposing that reduced IC_50_ values would indicate a greater oxidative stress in the plant caused by absorbed metal content.

### 2.5. Bioaccumulation Factors

The phytoextraction ability of *A. donax* L. plants was assessed using both the translocation factor (TF) and the bioaccumulation factor (BF) as follows.

(1) Translocation factor:
  (2) $$\begin{array}{c} \text{TF}=\frac{{\left[\text{Cd}\right]}_{\text{shoot}}}{{\left[\text{Cd}\right]}_{\text{root}}}. \end{array}$$
(2) Bioaccumulation factor:
  (3) $$\begin{array}{c} \text{BF}=\frac{{\left[\text{Cd}\right]}_{\text{shoot}}}{{\left[\text{Cd}\right]}_{\text{solution}}}. \end{array}$$

### 2.6. Analytical Procedure

The harvested plants were separated into stems, leaves, and branches and the fresh weight was recorded. For dry weight determination, plant material was oven-dried at 70°C for 24 h, weighed, ground with pestle and mortar, and sieved at 0.1 mm nylon sieve. Plant samples were digested through wet digestion for metals determination using the HNO_3_/HClO_4_. A 0.5 g sample was taken in 100 mL conical flask and 10 mL of Perchloric and Nitric acid mixture (1 : 2 ratios) was added to each conical flask and left overnight. Next day, glass funnels were placed at the mouth of each flask in such a way that funnel stem stayed at least one inch above the surface of liquid. The flasks were then placed on hot plate and the temperature was gradually increased to allow for effective digestion. It took about 15 to 20 minutes when HNO_3_ volatilized as nitrous oxide fumes, and then white fumes of Perchloric acid came out from the flask. The solution in flask was white in color at that stage. After digestion, the flasks were removed from the hot plate allowed to cool and few milliliters of distilled water was added. The digested material was then transferred to 50 mL volumetric flask and the volume was made up to 50 mL with deionized water. The readings were measured on Perkin Elmer Atomic Absorption Spectrometer-700 [15].

### 2.7. Soil Analysis

The soil used in the experiment was collected from an experimental field in COMSATS Institute of Information Technology, Abbottabad. The soil was sieved to remove roots, pebbles, and other unwanted materials. The soil was analyzed for various physic-chemical parameters according to [8] and presented in Table 2.

At the end of growing periods, soil samples from two replicates were oven-dried at 70°C. Soil sample (1 g) was digested on a hot plate with 15 mL nitric acid and 10 mL hydrogen peroxide. The digests were brought to 50 mL with deionized distilled water and the impurities were removed by filtration. A total Cd content was analyzed by Perkin Elmer Atomic Absorption Spectrometer-700.

### 2.8. Statistical Analysis

All determinations were performed in triplicate and mean values are presented in the results. One-way analysis of variance (ANOVA) was carried out for both the experiments separately using SAS 8.3 software (SAS Ins. Inc., Cary, USA). Treatment means were portioned using Least Significant Difference (LSD) at appropriates *α* value (0.05).

## 3. Results

### 3.1. Cadmium Content of Plant

**Figure 1:**
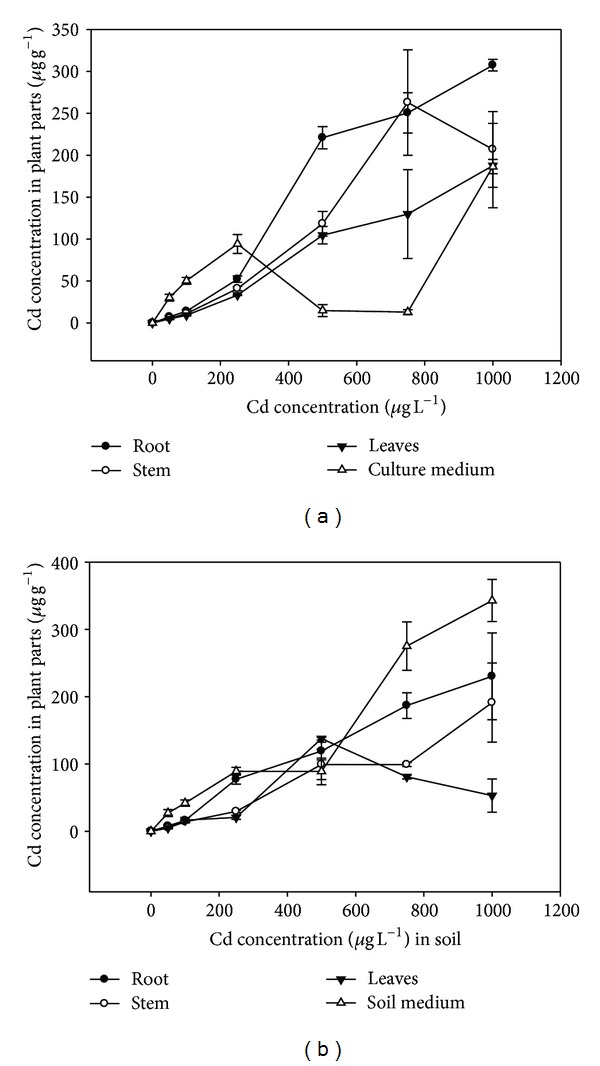
(a) The accumulation of cadmium in various plant parts in hydroponics experiment. (b) The accumulation of cadmium in various plant parts during soil experiment.

The results of the cadmium concentration in the different plant parts grown in contaminated water were presented in Figure 1(a). In root, the cadmium uptake had linear relation to the increasing supplied cadmium concentration. Root Cd contents at various treatments significantly (*P* < 0.05) differed. The maximum cadmium uptake in stem was noted at 750 *μ*g L^−1^ (262.8 *μ*g g^−1^). However, the cadmium concentration at 1000 *μ*g L^−1^ was significantly (*P* < 0.05) different from all other treatments except 750 *μ*g L^−1^. Cd accumulation in leaves was the maximum at 1000 *μ*g L^−1^ (187.5 *μ*g L^−1^) and was significantly (*P* < 0.05) different from the rest of the treatments. For the treatments 50 to 500 *μ*g L^−1^, the leaf cadmium content was in the range of 4.8 to 129.83 *μ*g g^−1^. Overall, the Cd accumulation pattern in various plant organs was as follows: root > stem > leaf (Figure 1(a)). The left over Cd content in the aqueous medium was in the range of 12 to 186.4 *μ*g L^−1^ for various treatments. The maximum left over cadmium concentration (186.4 *μ*g L^−1^) was noted for 1000 *μ*g L^−1^ supplied cadmium (Figure 1(a)).

The results of the cadmium concentration in plant parts grown in contaminated soil were presented in Figure 1(b). The maximum Cd concentration in roots of soil grown plants was 230 *μ*g g^−1^ at 1000 *μ*g g^−1^ supplied Cd content. The plant root Cd content was significantly (*P* < 0.05) different from supplied Cd contents of 500 *μ*g g^−1^ and below. For stem, the maximum cadmium uptake occurred at 1000 *μ*g g^−1^ which was 191 *μ*g g^−1^ which was significantly (*P* < 0.05). The stem cadmium concentration at 750 *μ*g L^−1^ was 99 *μ*g g^−1^, significantly (*P* < 0.05) different from the values at 250 *μ*g g^−1^ and lower supplied Cd treatments (Figure 1(b)). The maximum uptake in leaves of soil grown plants occurred at 500 *μ*g g^−1^ treatment which was 138 *μ*g g^−1^ and was significantly (*P* < 0.05) different from the rest of treatments. At higher treatments (750 to 1000 *μ*g L^−1^), the leaf cadmium contents were 81 and 53 *μ*g g^−1^, respectively, and were nonsignificantly different (*P* > 0.05). Like hydroponics, the accumulation pattern of cadmium in various plant organs was as follows: root > stem > leaf (Figure 1(b)). The left over cadmium concentration in the soil medium was in the range of 27 to 343 *μ*g g^−1^ soil for various supplied cadmium concentrations.

### 3.2. Effect of Cadmium on Photosynthetic Pigments

**Figure 2:**
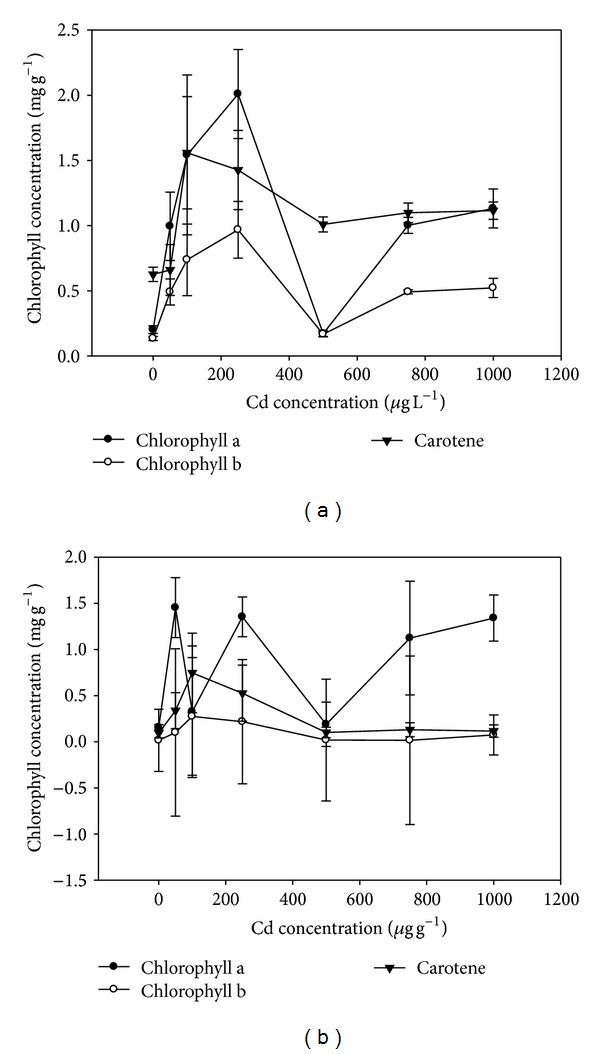
(a) The effect of cadmium concentrations on chlorophyll content in hydroponics experiment. (b) The effect of cadmium concentrations on chlorophyll content during soil experiment.

The effects of cadmium on the photosynthetic pigments that is chlorophyll a, chlorophyll b, and carotenes of plants grown in hydroponics were presented in Figure 2(a). The maximum chlorophyll a content was observed at Cd treatment of 250 *μ*g L^−1^ (Figure 2(a)). The amount of chlorophyll b had similar trend like chlorophyll a with the maximum value (0.96 mg g^−1^) at 250 *μ*g L^−1^ supplied Cd (Figure 2(a)). As far as carotene content was concerned, its amount was the maximum (1.55 mg g^−1^) at 100 *μ*g L^−1^. Further increase in the supplied Cd did not cause any significant (*P* < 0.05) increase in carotene content. However, the amount of carotene was significantly (*P* < 0.05) different than control and 50 *μ*g L^−1^ (Figure 2(a)).

The effects of cadmium on the photosynthetic pigments in soil grown plants were presented in Figure 2(b). The chlorophyll a content was the maximum at Cd treatment of 1000 *μ*g g^−1^. The amount of chlorophyll b increased up to 250 *μ*g g^−1^ where its value was 0.21 mg g^−1^ and then it showed a decline towards the maximum Cd content in soil. As far as carotene content was concerned, amount was the maximum (0.74 mg g^−1^) at 100 *μ*g g^−1^ supplied Cd in soil. Further increase in the supplied cadmium did not cause any increase in carotene content (Figure 2(b)).

### 3.3. Effect of Cadmium on Bioconcentration Factors

**Figure 3:**
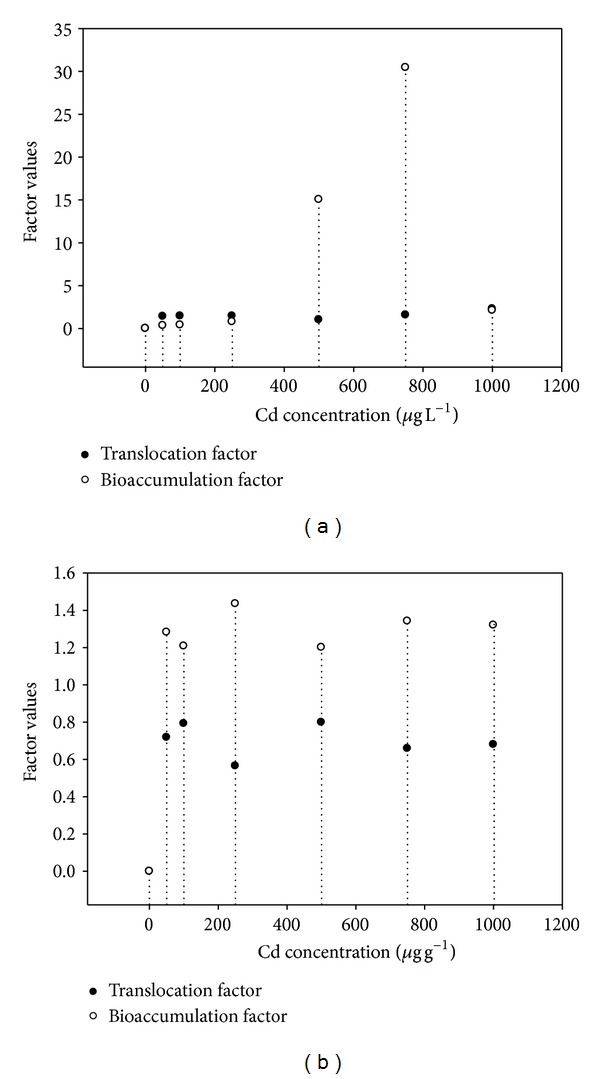
(a) Relative bioconcentration factors at various cadmium treatments in hydroponics. (b) Relative bioconcentration factors at various cadmium treatments in soil.

The results of bioconcentration factors for plants grown in hydroponics were presented in Figure 3(a). The translocation and bioaccumulation factors increased up to 750 *μ*g L^−1^ Cd in the hydroponics culture (Figure 3(a)). Overall, BF and TF values were in the range of 0.3 to 30 and 1 to 2.28, respectively, for various Cd treatments. The highest values were TF = 1.6 and BF = 30.00 for 750 *μ*g L^−1^ Cd treatment. However, both TF and BF decreased at 1000 *μ*g L^−1^ and some toxicity symptoms appeared in the plants receiving that Cd treatment (Figure 3(a)). Both bioconcentration factor values for soil grown plants were presented in Figure 3(b). The translocation and bioaccumulation factors increased as a function of Cd concentration up to 500 *μ*g g^−1^ supplied Cd; however, it decreased a little at higher Cd content in soil (Figure 3(b)). Overall, the values of BF and TF were in the range of 0.7 to 0.67 and 1.2 to 1.3, respectively, for various Cd treatments. The highest values were TF = 1.3 and BF = 0.71 for the Cd treatment of 500 *μ*g g^−1^. Translocation factors were above the reference value (1.0) for hyperaccumulation; however, BF values were below 1 (Figure 3(b)).

### 3.4. Effects of Cadmium on Growth Characteristics

**Figure 4:**
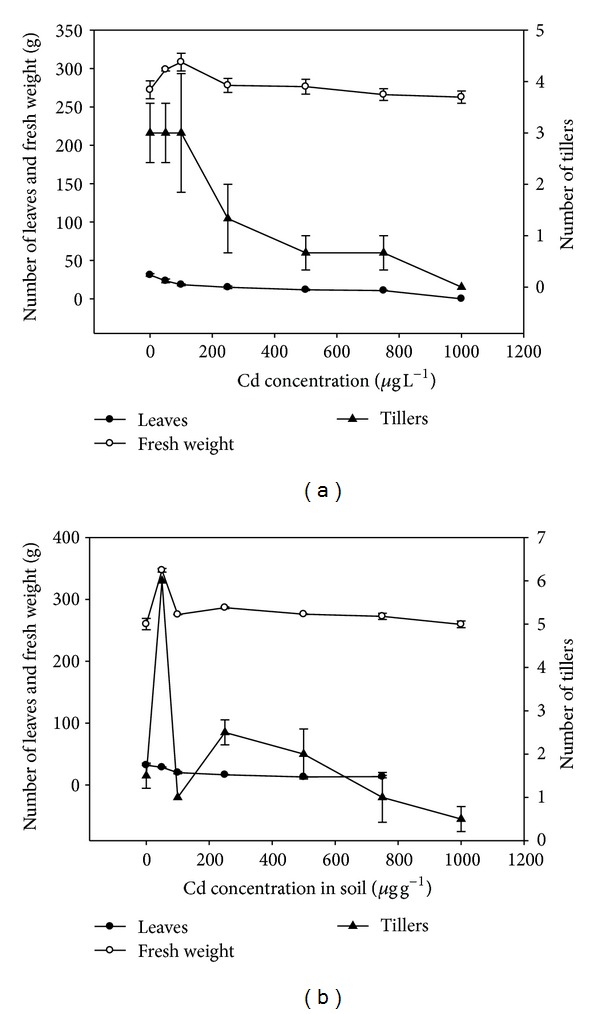
(a) Effect of cadmium on fresh weight, number of leaves, and tillers of treated plants growth in hydroponics culture. (b) Effect of cadmium on fresh weight, number of leaves, and tillers of treated plants growth in in soil.

Growth performance of the plant in reference to plant height, nodes, internodes, tillers, fresh weight, and number of leaves of plants from hydroponics is depicted in Figure 4(a). The results showed that there was no significant (*P* < 0.05) increase in plant height, root length, nodes and internodes of treated plants at all levels of Cd treatments. However some increase in the fresh weight, leaves, and tillers of the treated plants was observed. For leaves, the number significantly increased up to 100 *μ*g L^−1^ supplied Cd. The number was significantly different at all other treatments except 250 and 500 *μ*g L^−1^ supplied Cd level. It implied that Cd initially enhanced the growth of plants up to the concentration of 300 *μ*g L^−1^ in hydroponics culture; however, at higher concentrations, the growth of the plant was reduced (Figure 4(a)). Growth performance of the plant in reference to plant height, tillers, fresh weight, and number of leaves during the soil experiment is depicted in Figure 4(b). A trend of growth similar to hydroponics experiment was observed. The observation that increase in number of tillers without increase in plant height (stunted growth) indicated Cd stress.

### 3.5. Antioxidant Assays

**Figure 5:**
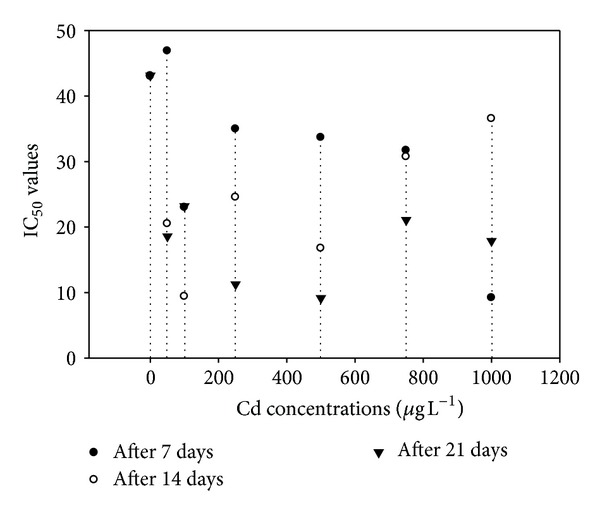
The effect of cadmium concentrations on the IC_50_ values against antioxidant activity of ABTS.

**Figure 6:**
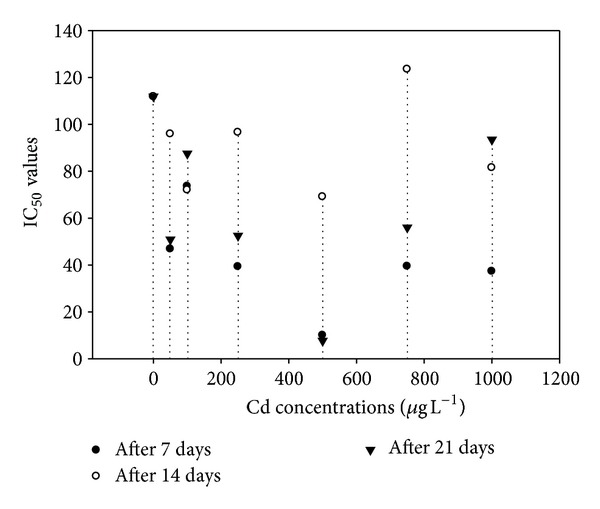
The effect of cadmium concentrations on the IC_50_ values against antioxidant activity of DPPH.

Antioxidants are chemical compounds that can bind to free oxygen radicals preventing these radicals from damaging healthy cells. The present study involved the determination of one of the most commonly used organic radicals for the evaluation of antioxidant efficiency of pure compounds and complex mixtures is the radical cations derived from ABTS and DPPH. These radical cations could be generated by enzymatic, chemical, and electrochemical means. The IC_50_ measured at 7th, 14th, and 21st days of the experiment were presented in Figures 5 and 6. The IC_50_ values of ABTS showed that both time and increasing Cd concentrations strongly inhibited the production of ABTS (Figure 5) which was indicated by lower IC_50_ value on 21st day. A similar trend was obvious for DPPH up to 500 *μ*g L^−1^ supplied Cd (Figure 6). However, a relative increase in IC_50_ was observed at higher supplied Cd contents (>500 *μ*g L^−1^) showing that the concentration of these antioxidants might be greater with increasingly higher Cd exposure.

## 4. Discussion

The present study employed the use of *A. donax* to treat Cd metal in hydroponics and soil contaminated environments. Overall, the results indicated that the plant is useful for the treatment of Cd contaminated wastewaters. However, better uptake was observed in hydroponics cultures as compared with soil environment. The maximum plant root Cd content was 300 *μ*g g^−1^ in hydroponics experiment as compared with 230 *μ*g g^−1^ in soil experiment. Likewise, Cd concentration in stem for hydroponics culture was 262 *μ*g g^−1^ at 750 *μ*g L^−1^ supplied Cd over 191.2 *μ*g g^−1^ at 1000 in soil experiment. In case of leaves, the maximum Cd concentration for hydroponics was 187 *μ*g g^−1^ at 1000 *μ*g L^−1^ supplied Cd. In soil experiment, the Cd concentration in leaves was 137 *μ*g g^−1^ at 500 *μ*g L^−1^. Relatively low Cd uptake occurred during soil experiment which consequently resulted in low TF values. It was opposite in case of hydroponics where BF and TF values were always greater than 1. Both factors values were above the reference value (1.0) for hyperaccumulation.

*A. donax* L. (giant reed, Poaceae) is a potentially high-yielding nonfood crop, which can be used for the production of energy, paper pulp, and wooden building materials [11]. It is a robust invasive perennial grass, wild growing in southern European regions and other Mediterranean countries [16]. It is also very common in Pakistan. Giant reed can easily adapt to different ecological conditions and grow in all types of soils [17]. The plant has been evaluated for the phytoremediation ability towards arsenic contamination [7]. It was suggested that *A. donax* plants may be employed to treat water containing arsenic concentrations up to 600 *μ*g L^−1^ [7].

Increasing metal concentrations in the wastewaters and soils adjacent to industrial regions of the world are a serious threat to the natural environmental sustainability. Among the heavy metals, cadmium is of special concern due to its potential toxicity to biota at low concentrations [18]. The use of indigenous plants like *A. donax* is very promising to combat metal toxicity in soils and wastewaters. Different plant species have different capacities for uptaking and tolerating the heavy metals like cadmium and others [19, 20]. The metal hyperaccumulators show an extra aptitude for accumulating the large quantity of metals in their aerial parts [21]. This special characteristic of the metal hyperaccumulators make them extremely appropriate for phytoremediation, that is, to use plants for cleaning up the polluted soils. In the preceding decade many studies have been accomplished to explore the mechanisms liable for the better metal uptake and tolerance via natural hyperaccumulators as model plant species [22]. In general the metal hyperaccumulation in plants is acknowledged as a mishmash of high metal uptake coupled with an improved tissue tolerance against the detrimental effects of higher metal concentrations through a better antioxidative response and sequestration at the cellular level [23]. Remediation of heavy metals contaminated that soil may possibly be carried out using physicochemicals processes such as ion-exchange, precipitation, reverse osmosis, evaporation, and chemical reduction; however, the procedures requisite external man-made resources and expensive [1].

Plants absorb toxic metals, translocate and accumulate them in roots and shoots; and finally resist to metal contamination, thus remediate contaminated environments [24]. Phytoremediation is a growing field of research in environmental studies because of the advantages of its environmental friendliness, safe, cost effectiveness, and the possibility of harvesting the plants for the extraction of absorbed contaminants such as metals that cannot be easily biodegraded for recycling among others [7]. Moreover, it is based on the ecological engineering principles. The most effective phytoremediation plants are those classified as hyperaccumulators [25] and accumulators. Hyperaccumulators are characterized based on four features. First, the concentration in the shoots (stems or leaves) of a hyperaccumulator should be 10,000 *μ*g g^−1^ for Zn and Mn; above 1000 *μ*g g^−1^ dry mass for As, Pb, Cu, Ni, and Co; 100 *μ*g g^−1^ for 1 Cd and 1 *μ*g g^−1^ for Au [7, 8, 26]. Second is translocation factor (concentration in shoots/roots >1), metal concentrations in the shoots of a plant should be higher than those in the roots [7]. Third is bioaccumulation factor (concentration in plant/habitat >1) [7] and lastly it is tolerance ability, a hyperaccumulator should have high tolerance to toxic contaminants. *A. donax* L. can tolerate arsenic concentrations up to 600 *μ*g L^−1^ without toxicity symptoms appeared on the plant. The appearance of some toxicity symptoms in the leaves, roots, and slow growth at 1000 *μ*g L^−1^ revealed that although *A. donax* L. cannot tolerate but still accumulate and volatilize as concentration above 600 *μ*g L^−1^ [7–9].

The results suggested that Cd contamination affected the photosynthetic pigments to some extent. Detailed studies indicate that heavy metals have effects on photosynthetic pigments in plants. Heavy metals are known to interfere with chlorophyll synthesis either through direct inhibition of an enzymatic step or by inducing deficiency of an essential nutrient [27]. An important indicator which determines photosynthesis intensity is chlorophyll content in plant leaves. Cadmium markedly suppresses chlorophyll accumulation in leaves [28]. Carotenoid actively participates in photosynthesis as well and it was shown that content and ratio of carotenoids are strictly changed under impact of different stresses [29]. However, it has been determined that carotenoids are less sensitive to the impact of cadmium as compared to chlorophylls [30]. The success of phytoextraction is inherently dependent on several plant characteristics, the two most important being the ability to accumulate large quantities of biomass rapidly and the capacity to accumulate large quantities of environmentally important metals in the shoot tissue [31]. Effective phytoextraction requires both plant genetic ability and the development of optimal agronomic practices, including (1) soil management practices to improve the efficiency of phytoextraction and (2) crop management practices to develop a commercial cropping system. The present study showed that *A. donax* has potential to remediate the Cd contaminated environments as indicated by its high tissue Cd concentrations and BF and TF values higher than 1 especially for hydroponics experiment. The reason behind less Cd uptake in soil experiment may be that it may have been adsorbed in soil particles or it is leaching from rhizosphere as the experiment was conducted in sandy soil.

The IC_50_ value was defined as the concentration of the sample necessary to cause 50% inhibition, which was obtained by interpolation from linear regression analysis [32]. A lower IC_50_ value is associated with a higher radical scavenging activity. The IC_50_ values of ABTS showed that both time and increasing Cd concentrations affected the production of ABTS which was indicated by lower IC_50_ value on 21st days. A similar trend was obvious for DPPH upto 500 *μ*g L^−1^ supplied Cd. However, a relative increase in IC_50_ was observed at higher supplied Cd contents (>500 *μ*g L^−1^) showing that the concentration of these antioxidants might be greater with increasingly higher Cd exposure. One of the most commonly used organic radicals for the evaluation of antioxidant efficiency of pure compounds and complex mixtures is the radical cation derived from 2,2′-azinobis-3-ethyl-benzothiazoline-6-sulfonic acid (ABTS) [33]. The differences among all studied morphological parameters were statistically nonsignificant; likewise, the fresh and dry weights of control and experimental plants were also nonsignificant. Reduced growth was noted in control plants which may possibly be due to the fact that the plants were grown in pots and not in the field. *A. donax* is a grass with C3 photosynthetic pathway unlike other grasses (e.g., switchgrass and miscanthus) with C4 pathway [34]. Physiological processes, such as photosynthesis and water status, are sensitive to heavy metals [35] in several plant species. Heavy metals have been found to inhibit electron transport in photosynthetic systems [36]. Photosynthetic rates of *A. donax* were unaffected by the treatments, indicating that the photosynthetic system was not harmed and showed a strong tolerance of this plant to the increased heavy metal concentrations in the soil. The mean values of giant reed Pn rates found in this study were higher than those usual for C3 plants (18–20 *μ*mol CO_2_ m^−2^ s^−1^) [37]. Rossa et al. [38], in a comparative study on photosynthesis of five C3 and three C4 grasses, found that *A. donax* had high Pn rates, higher than the other grasses (37.0 *μ*mol CO_2_ m^−2^ s^−1^) under similar environmental conditions.

## 5. Conclusions

The phytoremediation ability of *Arundo donax* to treat cadmium contamination was compared in hydroponics and soil environments. The plant is useful for the treatment of Cd contaminated wastewaters both in hydroponics and in soil environments. However, better uptake was observed in hydroponics cultures as compared with soil environment. Both BF and TF values for hydroponics were greater than 1 which confirmed its suitability for aquatic contaminated environments. At higher Cd exposure, plant showed some antioxidative stress as the concentration of antioxidants was greater with increasing Cd exposure.

## Figures and Tables

**Table 1:** Average values of various morphological parameters before cadmium treatment.

Parameters	Values
Plant height (cm)	55 ± 12
No. of leaves per plant	87 ± 18
No. of nodes per plant	33 ± 22
Average root length per plant (cm)	8 ± 2
Toxicity symptoms	Leaf burning

**Table 2:** Physicochemical properties of the soil used in experiment.

Characteristic	Value
pH	6.8
Available nitrogen (mg kg^−1^)	75.4
Available potassium (mg kg^−1^)	62.1
Available phosphorus (mg kg^−1^)	7.8
Total nitrogen (g kg^−1^)	0.86
Total phosphorus (g kg^−1^)	0.7
Total potassium (g kg^−1^)	17.3
Organic matter (g kg^−1^)	21.4
